# The durability of long-lasting insecticidal nets distributed to the households between 2009 and 2013 in Nepal

**DOI:** 10.1186/s41182-020-00223-w

**Published:** 2020-05-19

**Authors:** Prakash Ghimire, Komal Raj Rijal, Nabaraj Adhikari, Garib Das Thakur, Baburam Marasini, Upendra Thapa Shrestha, Megha Raj Banjara, Shishir Kumar Pant, Bipin Adhikari, Shyam Prakash Dumre, Nihal Singh, Olivier Pigeon, Theeraphap Chareonviriyaphap, Irwin Chavez, Leonard Ortega, Jeffrey Hii

**Affiliations:** 1 Central Department of Microbiology, Tribhuvan University, Kathmandu, Nepal; 2 World Health Organization Country Office for Nepal, Pulchowk, Lalitpur, Nepal; 3 Epidemiology and Disease Control Division, Department of Health Services, Ministry of Health and Population, Kathmandu, Nepal; 4 VectorBorne Disease Research and Training Center, Ministry of Health and Population, Hetauda, Nepal; 5 Centre for Tropical Medicine and Global Health, Nuffield Department of Medicine, University of Oxford, Oxford, UK; 6 Department of Immunogenetics, Institute of Tropical Medicine (NEKKEN), Nagasaki University, Nagasaki, Japan; 7 Agriculture and Natural Environment Department, Plant Protection Products and Biocides Physico-chemistry and Residues Unit, Walloon Agricultural Research Centre (CRA-W), Carson Building, Rue du Bordia, 11, B-5030 Gembloux, Belgium; 8 Department of Entomology, Kasetsart University, Bangkok, Thailand; 9 Department of Tropical Hygiene, Faculty of Tropical Medicine, Mahidol University, 420/6 Rajvithi Road, Bangkok, 10400 Thailand; 10 South-East Asia Regional Office, World Health Organization, New Delhi, India; 11 Global Malaria Programme World Health Organization, 20 Avenue Appia, 1211 Geneva 27, Switzerland; 12 Malaria Consortium, Faculty of Tropical Medicine, Mahidol University, 420/6 Rajvithi Road, Bangkok, 10400 Thailand; 13 College of Public Health, Medical & Veterinary Sciences, James Cook University, Townville, QLD 4811 Australia

**Keywords:** Long-lasting insecticide treated nets, Durability, Malaria, Bio-efficacy, Chemical retention, Proportionate hole index, Nepal

## Abstract

**Background:**

Understanding and improving the durability of long-lasting insecticidal nets (LLINs) in the field are critical for planning future implementation strategies including behavioral change for care and maintenance. LLIN distribution at high coverage is considered to be one of the adjunctive transmission reduction strategies in Nepal’s Malaria Strategic Plan 2014–2025. The main objective of this study was to assess the durability through assessment of community usage, physical integrity, residual bio-efficacy, and chemical retention in LLINs: Interceptor®, Yorkool®, and PermaNet ®2.0 which were used in Nepal during 2009 through 2013.

**Methods:**

Assessments were conducted on random samples (*n* = 440) of LLINs from the eleven districts representing four ecological zones: Terai plain region (Kailali and Kanchanpur districts), outer Terai fluvial ecosystem (Surkhet, Dang, and Rupandhei districts), inner Terai forest ecosystem (Mahhothari, Dhanusa, and Illam districts), and Hills and river valley (Kavrepalanchock and Sindhupalchok districts). For each LLIN, fabric integrity in terms of proportionate hole index (pHI) and residual bio-efficacy were assessed. However, for chemical retention, a representative sample of 44 nets (15 Yorkool®, 10 Permanet®2.0, and 19 Interceptor®) was evaluated. Data were analyzed using descriptive statistics stratified by LLINs brand, districts, and duration of exposure.

**Results:**

On average, duration of use of LLINs was shortest for the Yorkool® samples, followed by PermaNet® 2.0 and Interceptor® with median ages of 8.9 (IQR = 0.4), 23.8 (IQR = 3.2), and 50.1 (IQR = 3.2) months, respectively. Over 80% of field distributed Yorkool® and PermaNet® 2.0 nets were in good condition (pHI< 25) compared to Interceptor® (66%). Bio-efficacy analysis showed that average mortality rates of Interceptor and Yorkool were below World Health Organization (WHO) optimal effectiveness of ≥ 80% compared to 2-year-old PermaNet 2.0 which attained 80%. Chemical retention analysis was consistent with bio-efficacy results.

**Conclusion:**

This study shows that distribution of LLINs is effective for malaria control; however, serviceable life of LLINs should be considered in terms of waning residual bio-efficacy that warrants replacement. As an adjunctive malaria control tool, National Malaria Control Program of Nepal can benefit by renewing the distribution of LLINs in an appropriate time frame in addition to utilizing durable and effective LLINs.

## Background

Malaria is endemic in the Terai region of Nepal that shares a long and porous border with India. Nepal is currently at the pre-elimination stage and targets to eliminate the malaria by 2025 [1, 2]. More than 80% of malaria infections in Nepal are caused by the relapsing parasite *Plasmodium vivax* [3]*.* Malaria is endemic in 65 of Nepal’s 77 districts, and thirteen districts are considered to be highly endemic by the National Malaria Control Program of Nepal (NMCP) [1–3]. One fifth of the *P. vivax* strains circulating in Nepal comprises long latency strain although currently recommended radical treatment of acute vivax malaria in Nepal with chloroquine (CQ), and a standard 14-day course of primaquine (PQ) is highly efficacious in preventing both short and long latency relapses [4, 5].

Nepal’s geographical landscape provides a unique relevance to malaria cases and the vectors [3]. The country has three ecological zones that run east to west: the Terai or lowland plains with a subtropical climate; the hill zone, which is more temperate; and the mountain zone, with an alpine climate [3, 6, 7]. Historically, malaria cases have largely been confined to the Terai, which is home to over half of Nepal’s total population and shares a southern border with India [2].

Amongst vectors transmitting malaria, *Anopheles minimus* was one of the important and highly efficient vectors in forest and forest fringe areas, but was reported to be eliminated following DDT spraying in the 1960s [8]. Over the last decades, *Plasmodium vivax* has predominated malaria transmission in Nepal, which is highly seasonal with the majority of transmission occurring from June to September [3, 8]. The burden of malaria is high amongst population from ethnic minorities and lower socio-economic status, with higher number of cases in young men, and mobile and migrant populations of border areas [9, 10].

Multipronged strategies are currently underway in Nepal to achieve the goal of malaria elimination. In line with these efforts, Nepal’s Ministry of Health and Population (MoHP) with the support of Global Fund and WHO has been implementing a malaria control programme that includes Insecticide Residual Spraying (IRS) in selective high-risk areas together with distribution of long-lasting insecticidal nets (LLINs) in high and moderate risk areas. LLINs are distributed based on the national malaria strategic plan that entails one LLIN per two persons in a household [11]. Vector control is an integral component of the NMCP. Apart from IRS, LLINs are distributed to populations living in high risk areas within an administrative division referred as Village Development Committees (VDCs). Pregnant women in high- and moderate-risk VDCs are given an additional net during antenatal care (ANC) visits [8, 12]. LLINs are distributed through a campaign with the strategy referred as “target one-third” which involves distributing LLINs to one third of target VDCs within a district per year, covering the entire high-risk VDCs by the end of the third year. Two rounds of routine IRS are carried out annually in each high-risk VDC unless LLIN population coverage in that VDC exceeds 80%. The first round is undertaken in May–June and the second round in August–September.

The significant decline of confirmed malaria including *P. vivax* malaria during 2003 to 2012 has coincided with the scale up of free LLIN distribution policy together with the adoption of artesunate-lumefantrine as the first line of treatment for uncomplicated confirmed *P. falciparum* malaria [3, 13]. IRS is gradually replaced by LLIN coverage in malaria-risk districts [9]. A previous unpublished report by Epidemiology and Disease Control Division (EDCD) mentioned that local villagers in Nepal generally wash clothes and LLINs with detergent powder and soap, and dry them in sunlight for 4–5 h. In 2009, bioassay tests conducted in Kanchanpur district have reported that LLINs were washed four times, and these LLINs showed a sharp decline in mosquito mortality by up to 58% [14]. The report concluded that LLIN distributed in 2010 had many public comments such as “it was weak,” “not durable,” and “not effective like previous LLIN.” In fact, there is considerable variation in LLIN durability due to contributing factors such as socioeconomic status, the house environment, and human behavior related to its use, including handling and washing of LLINs [15].

The bioassay study on LLINs conducted by the NMCP reported low optimal effectiveness having a mortality rate of ≤ 80% [15]. Based on this observation of sub-optimal durability of LLINs, the current study was mandated by the NMCP in collaboration with the WHO. The objective of this cross-sectional study was to assess the durability of three LLINs (Interceptor®, Yorkool®, and PermaNet®2.0) in terms of physical integrity, residual bio-efficacy, and chemical retention. These nets were distributed in 11 malaria endemic districts between 2009 and 2013.

## Methods

### Study area and population

The study was conducted in 11 districts: Kanchanpur, Kailali, Surkhet, Dang, Rupendeli, Mahottari, Dhanhusha, Ilam, Sindhuli, Kavre, and Sindhupalchowk, as shown in the map elsewhere [12]. The study sites represent the unique environment and cultural settings in which LLINs were distributed during the mass campaign between 2009 and 2013 at 100% coverage. Selected sites were chosen based on criteria such as epidemiology (annual malaria incidence > 0.01 per 1000 population from 2009 to 2011 and accessibility) [12].

Approximately, 4 million LLINs were distributed to host communities across Nepal as a part of district wide campaign. Interceptor® (BASF, Ludwigshafen, Germany) and PermaNet®2.0 (Vestergaard Fransden, Lausanne, Switzerland) LLINs were polyester 75D (PET-75D). Yorkool® (Tianjin Yorkool International Trading Co, Ltd, China) LLINs were polyester 100D (PET-100D). The LLINs were marked with identification numbers and distributed to all households through District Public Health Offices and its community networks. All households received malaria prevention education to inform its optimal use. In the following weeks, each household was visited by a team member to ensure that all LLINs were hung above sleeping areas for the optimal use.

### Sampling

A simple random sampling was employed following World Health Organization Pesticide Evaluation Scheme (WHOPES) guidelines [15]. Sample size was determined following the WHOPES criteria that recommended a sample of 40 nets per each brand of the LLIN in a district [15]. A sample of 40 LLINs per VDC was considered optimal. This was based on the assumption of one measurement per LLIN in each time point allowing an alpha error of 0.05, power of 80%, and standard deviation of 8.0 [16, 17]. The district and VDC-wise list of households receiving LLINs were obtained from the Epidemiology and Disease Control Division (EDCD). In a VDC, the first household was selected randomly using lottery method, and every alternate house from the list of households was selected for rest of the 39 households. All LLINs of selected household were examined.

All villages from the eleven districts that fulfilled the following criteria were included in the sampling frame: (a) villages listed in the top 10 with the highest numbers of malaria cases in 2014; (b) villages which had at least 50 households and had received LLINs in 2010 to 2014; (c) villages which were accessible and consented to participate; and (d) villages with universal coverage of LLINs. All samples were assessed for physical condition and residual bio-efficacy. Due to high costs for chemical analysis, a convenience sample of 10% (*n* = 44) of the total sample was selected for chemical content analysis.

### Data collection

After the completion of training for the field teams, household visits were conducted by a trained data collector during January to March 2014. After receiving informed verbal consent, the data collector conducted a household survey using pre-tested structured questionnaires with the head of the household to collect information on duration of LLIN use. In addition, the data collector examined the physical condition of the LLIN by assessing number and size of holes. The number of holes in the torn nets was categorized by size as small (0.5–< 2 cm), medium (2–10 cm), and large (> 10 cm) [15].

Residual bio-efficacy of field distributed LLINs was assessed at the Department of Entomology, Kasetsart University, Bangkok, Thailand, using cone bioassay tests following the standard WHOPES guidelines [18, 19]. Bioassays were conducted at 27 ± 2 °C and 80 ± 10% relative humidity. An insecticide susceptible laboratory colony of *Aedes aegypti* (United States Department of Agricultural-USDA strain) colonized in the Department of Entomology, Kasetsart University was used for contact bioassay tests. For each cone assay, five non-blood fed, 2- to 5-day-old female mosquitoes were exposed for 3 min in each cone and then held for 24 h with access to a sugar solution. For each sample, four replicates were performed. Knockdown and mortality rates were measured twice at 60 min and 24 h after exposure, respectively. A negative control from an untreated net was included in each round of cone bioassay testing. If the mortality in the control was > 10%, all the tests for that day were repeated [20].

A subsample of LLINs (*n* = 44) were assessed for insecticide (chemical) retention at Walloon Agricultural Research Centre (CRA-W), Gembloux, Belgium, using analytical methods based on standard reference CIPAC (Collaborative International Pesticide Analytical Council) methods. Briefly, deltamethrin and in its non-relevant impurity deltamethrin *R*-alpha isomer were extracted from PermaNet®2.0 and Yorkool® samples by refluxing for 30 min with xylene, and the content was determined by high performance liquid chromatography with UV diode array detection (HPLC-DAD) using dicyclohexyl phthalate as internal standard. Alpha-cypermethrin was extracted from Interceptor® samples by refluxing for 30 min with xylene in presence of citric acid, and the content was determined by gas chromatography with flame ionization detection (GC-FID) using dioctyl phthalate as an internal standard. These analytical methods were successfully validated on their specificity, linearity of chromatographic response, repeatability, reproducibility, and accuracy, and CRA-W has obtained the ISO 17025 accreditation for these methods. Analysis was carried out on four fabric samples (25 × 25 cm), and results were combined to provide the average concentration of the insecticide in each LLIN. Before analysis, each sample was kept in an aluminum foil at room temperature under shelter away from direct sunlight. Results were expressed in “g” active ingredient (a.i.)/kg and converted to mg a.i./m^2^ (mass of net in gram per m^2^) using the fabric weight. Accuracy and reproducibility of the analytical method were assessed by a concurrent replicate analysis of quality control samples for all LLIN brand samples.

### Data analysis

Data management and statistical analyses were performed using STATA 12.1 (StataCorp LP, College Station, TX, USA). Descriptive statistics were used to summarize the characteristics of households and variables of interest. Data entries from three data sources (household survey, bio-efficacy analysis, and chemical retention analysis) were consolidated before analysis. Duplicates in any of the records were excluded. Data from bio-efficacy analysis and chemical retention analysis were excluded if records did not match with LLIN registration numbers. Data were analyzed using descriptive statistics to summarize and compare between LLIN brands, districts, and duration of exposure (longer than/ less than or equal to 3 years). The following outcome measures were explored.

Proportionate hole index (pHI) as a measure for physical integrity. The pHI was calculated as recommended by WHO [15]. Data from the physical examination during the household visit were transformed into the pHI using the formula:

pHI = (No. size 1 hole × *A*) + (No. size 2 holes × *B*) + (No. size 3 holes × *C)* + (No. size 4 holes × *D*). The weights: *A* = 1, *B* = 23, *C* = 196, and *D* = 576 correspond to the areas estimated on the assumption that the hole sizes in each category are equal to the mid-points.

In addition, each net was then categorized as “good” (pHI< 65), “acceptable” (pHI = 65–643), or “torn” (pHI> 643) based on the WHO guidelines [15].

24 h mortality rate and 60 min knock down rates (KDR) are measures for residual bio-efficacy. If the mortality in the control was between 5 and 20%, the data were adjusted with Abbott’s formula [15]. Based on the WHO guideline, optimal effectiveness was further defined by target of functional mortality being ≥ 80% and target for KDR being ≥ 95% [19].

Chemical retention was measured as percent of actual content against baseline nominal active ingredient (a.i.) in g/kg. Baseline nominal chemical content for Yorkool® was 1.4 g/kg deltamethrin, PermaNet®2.0 was 1.8 g/kg deltamethrin; and Interceptor® was 6.7 g/kg alpha-cypermethrin. A tolerance limit of ± 25% of the baseline nominal content is recommended in the WHO specifications for unused LLINs [20–23].

## Results

### Sample

**Fig. 1:**
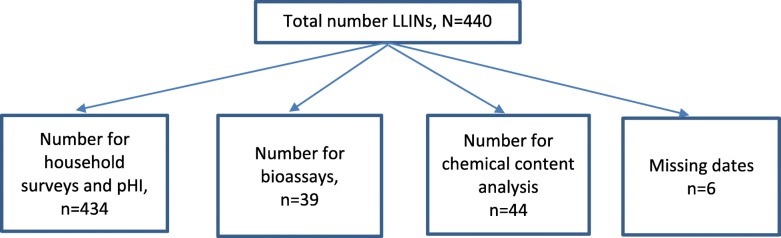
Flow chart of LLIN records collected for household, pHI, bioassays, and chemical analysis

The survey included 440 randomly selected households. Six of the original 440 LLINs were excluded because of incomplete data for key variables; these comprised three Interceptors, two PermaNet® 2.0, and one Yorkool® LLINs. Of a total of 440 LLINs, 434 were examined during household and pHI surveys, 329 bioassayed for residual efficacy, and 44 were chemically analyzed (Fig. 1).

The proportional breakdown of the 11 surveyed districts was Kailali (9.1%), Kanchanpur (9.1%), Surkhet (11.4%), Dang (11.4%), Rupandehi (11.4%), Mahottari (9.1%), Dhanusa (6.8%), Ilam (9.1%), Sindhuli (9.1%), Kavre (6.8%), and Sindupalchowk (6.8%) (Table 1). The surveys covered a total of 440 households (56.8% in high and 43.2% in medium risk strata) among 22 VDCs.

**Table 1:** Number of field-distributed nets sampled per campaign brand by district

	Number of nets (percent of total)				
Ecozone	District	Interceptor®	PermaNet 2.0	Yorkool	Total
Terai plain rice	Kailali	20	0	20	40
	Kanchanpur	39	0	1	40
Outer terai foothill fluvi ecosystem	Surkhet	0	22	28	50
	Dang	0	25	25	50
	Rupendeli	0	25	25	50
Inner terai forest ecosystem	Mahottari	34	1	5	40
	Danusha	25	0	5	30
	Ilam	25	0	15	40
Hills and river valley	Sindhuli	36	1	3	40
	Kavre	21	2	7	30
	Sindupalchowk	0	14	16	30
Total		200	90	150	440

On average, duration of use of LLINs was shortest for the Yorkool® samples, followed by PermaNet® 2.0 and Interceptor® with median age of 8.9 (IQR 0.4), 23.8 (IQR 3.2), and 50.1 (IQR 3.2) months, respectively. The differences are statistically significant with *p* < 0.001 derived from a Kruskal-Wallis test. Median duration of usage of LLINs by campaign brand is presented in Table 2.

**Table 2:** Average duration of community usage and by effectiveness of LLIN brands

	Interceptor®	PermaNet® 2.0	Yorkool®	
*N*	165	62	102	*p*
Average duration of community usage in months (95 % CI)	44.9 (42.6, 47.4)	24.8 (22.6, 26.9)	9.3 (8.6, 9.9)	
Median duration of community usage in months (IQR)	50.1 (3.2)	23.8 (3.2)	8.9 (0.4)	< 0.001^*^
More/less than or equal to 3 years of community usage, number of nets (percent of campaign sample)	≤ 3 years: *n* = 24 (14.5) > 3 years: *n* = 141 (85.5)	≤3 years: *n* = 61 (98.4) >3 years: *n* = 1 (1.6)	≤3 years: *n* = 102 (100) >3 years: *n* = 0 (0)	
Median age of nets with > 80% mosquito mortality (IQR)	50.3 (6.1)	23.8 (3.2)	9.2 (0.9)	< 0.001^*^
Percent nets bioassayed with > 80% mosquito mortality (n)	9.7 (*n* = 16)	53.2 (*n* = 33)	9.8 (*n* = 10)	

*Kruskal-Wallis ANOVA; level of significance = 0.05

### Proportionate hole index (pHI)

**Fig. 2:**
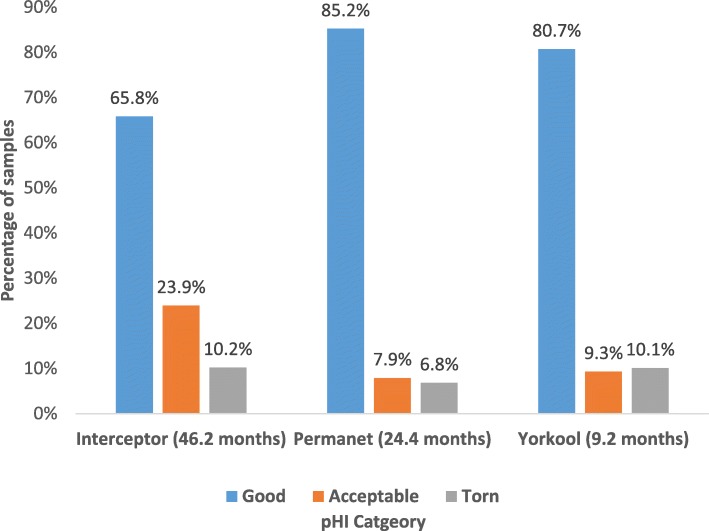
Distribution of proportionate hole index (pHI) category by LLIN brand

Physical conditions of all three LLIN brands varied with age. Yorkool® had the shortest community usage of 9.2 months. Of 150 Yorkool® nets, 80.7% (*n* = 121) were in good condition, 9.3% (*n* = 14) were in acceptable condition, and 10.1% (*n* = 15) of nets were torn. Of 88 PermaNet® 2.0 nets, 85.6% (*n* = 75) were in good condition, 6.7% (*n* = 7) were in acceptable condition, and 6.8% (*n* = 6) of nets were torn. Of 197 field-distributed Interceptor® nets, 65.8% (*n* = 132) were in good condition, 23.9% (*n* = 47) were in acceptable condition, and 10.2% (*n* = 20) of nets were torn (Fig. 2).

### Residual bio-efficacy

**Fig. 3:**
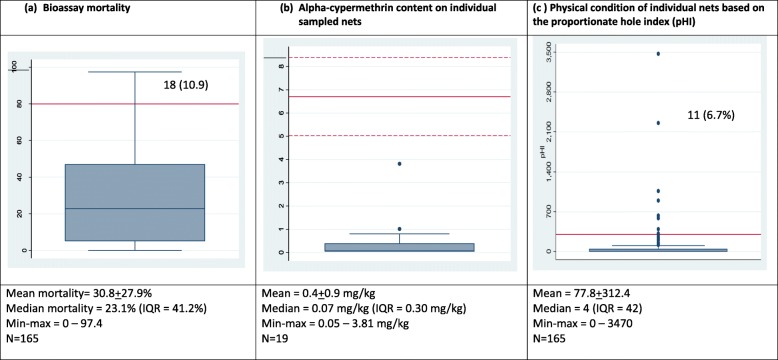
Boxplot of Interceptor LLIN showing **a** median mortality, inter-quartile range (0.25–0.75), and max-min range. Full red line is the 80% mortality threshold. **b** Target dose of alpha-cypermethrin (full red line) and tolerance limits (± 25% of target dose, dashed lines (**b**), and (**c**) mean pHI threshold for replacement of nets (full red line. Nos (%) indicate the number of data points (percent)

**Fig. 4:**
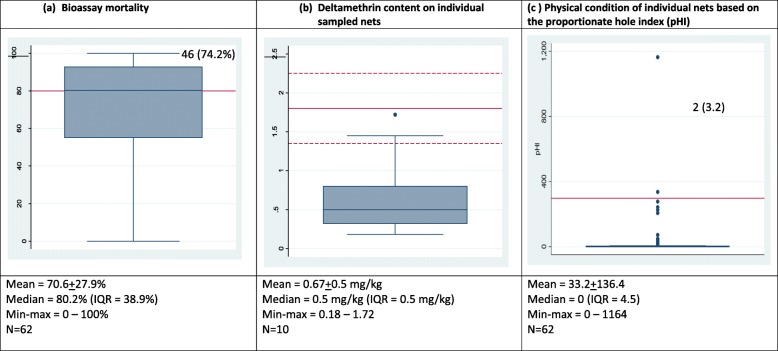
Boxplot of PermaNet 2.0 2.0 showing **a** median mortality, inter-quartile range (0.25–0.75), and max-min range. Full red line is the 80% mortality threshold (**a**), target dose of deltamethrin (full red line), and tolerance limits (± 25% of target dose, dashed lines (**b**), and (**c**) mean pHI threshold for replacement of nets (full red line). Nos (%) indicate the number of data points (percent) above the cutoff red lines

**Fig. 5:**
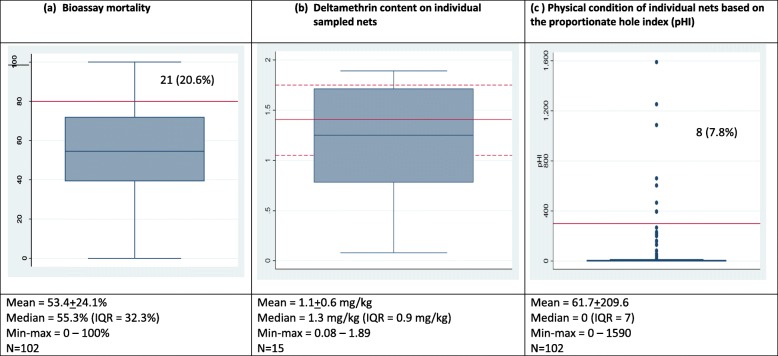
Boxplot of Yorkool showing **a** median mortality, inter-quartile range (0.25–0.75), and max-min range. Full red line is the 80% mortality threshold (**a**), target dose of deltamethrin (full red line), and tolerance limits (± 25% of target dose, dashed lines (**b**), and (**c**) mean pHI threshold for replacement of nets (full red line). Nos (%) indicate the number of data points (percent) above the cutoff red lines

Residual bio-efficacy tests were observed for 197 (45.4%) of Interceptor®, 149 (34.3%) of Yorkool®, and 88 (20.3%) of PermaNet® 2.0 nets. As the knockdown rates of *Ae. aegypti* were low (0.88% Interceptor®, 0.14% Yorkool® and 1.28% PermaNet® 2.0, data not shown), mosquito mortality rate was the preferred indicator of bio-efficacy. There was no mortality in the control tests among 1760 bioassay tests (data not shown). Median mortality rate was the highest for 2-year-old PermaNet® 2.0 nets (80.2%; IQR = 38.9%) (Fig. 4a), followed by Yorkool® (55.3%; IQR = 32.2%) (Fig. 5a) and the lowest for the oldest sample of Interceptor® nets (23.1%; IQR = 41.2%) (Fig. 3a). The percentage of PermaNet® 2.0 (*n* = 33), Yorkool® (*n* = 10), and Interceptor® (*n* = 16) nets passing the > 80% mortality cutoff was 53.2%, 9.8%, and 9.7%, respectively (Table 2).

### Chemical retention

A total of 44 nets comprising 19 Interceptor®, 10 PermaNet® 2.0, and 15 Yorkool® LLINs were chemically tested (Table 3). The average active pyrethroid content of each of the three LLIN brands varied from 0.39 mg/kg alpha-cypermethrin (Interceptor®) to 1.15 mg/kg deltamethrin (Yorkool®) (Figs. 3b, 4b, and 5b). The average active deltamethrin and alpha-cypermethrin content of PermaNet®2.0 (Fig. 4), Yorkool (Fig. 5), and Interceptor® (Fig. 3) measured over the entire > 24 months post distribution did not reach the given target dose. None of the samples (within net RSD = 223.7%) taken from Interceptor nets were within the limits of the target dose, while 20% (within net RSD = 80.1%) of Permanet®2.0 and 60% (within net RSD = 50.6%) of Yorkool® samples were within their respective target doses (Table 3).

**Table 3:** Mean chemical content and retention of active ingredient (AI) by LLIN brand

	Interceptor	PermaNet 2.0	Yorkool
Active ingredient	Alpha-cypermethrin	Deltamethrin	Deltamethrin
Number of nets	19	10	15
Mean age of nets (months)	46.2	24.4	9.2
Mean chemical content (g/kg), 95% CI^b^	0.39 (0.005–0.879)	0.67 (0.28–1.04)	1.15 (0.005–0.879)
Relative standard deviation, %	223.7	80.1	50.6
% mean retention of AI	5.82	47.9	85.2
% nets within the tolerance limits of the target dose	0%	20%	60%

^a^The target doses at baseline (tolerance limits of 25%) are 6.7 (5.025–8.375) g/kg, 1.4 (1.05–1.75) g/kg, and 1.8 (1.35–2.25) g/kg for Interceptor, Yorkool, and PermaNet 2.0 respectively. Within net variation is expressed by the relative standard deviation (RSD%)

^b^The mean insecticide concentration was not significantly different between Yorkool, Interceptor, and PermaNet 2.0 (*p* > 0.05)

## Discussion

### Overview of findings

This study evaluated the performance of three brands of LLINs (Yorkool®, PermaNet® 2.0, and lnterceptor®) based on the WHO guidelines with a median age of 8.9, 23.8, and 50.1 months, respectively, in 11 malaria endemic districts of Nepal. The results from this study have several implications for the procurement and distribution of LLINs by NMCP. The proportion (90.9%) of the remaining Interceptor® nets in need of replacement, after 3 years, was large enough to suggest that the intervention would lose impact after the third year of the distribution-replacement cycle and after the first year for Yorkool® nets (proportion of 87.9%). Half of the sampled Permanet® 2.0 nets were still efficacious after an average of 2 years in use (Table 2). The percent mortality induced by Interceptor® was lower than WHO’s recommendation of 80% cutoff. A major limitation of the study was the lack of robust data of a cohort of nets that are monitored annually for up to 3 years or more. Secondly, the study lacked comparative data with a new Interceptor® LLIN of similar age. This information may be relevant to the NMCP as the effectiveness of LLIN brand will affect future mass or continuous distribution campaigns. A prospective $100,000 durability study is as cost-effective as rapid diagnostic tests (RDTs) when implemented as part of every one million net procurement system which is expected to cost less than 1% of total commodity costs [24].

### Efficacy of LLINs: Interceptor®, Yorkool® and PermaNet® 2.0

Polyester-based LLINs with 2 years of usage, such as Permanent®2.0, showed comparatively better quality based on bioassay tests compared to less than 1-year-old Yorkool®; however, an inverse relationship between bioassay mortality and chemical retention was seen with Permanent®2.0 and Yorkool®. Integrating the results of pHI, bio-efficacy, and chemical analysis showed that Interceptor® LLINs with more than 3 years of usage were inferior compared to Yorkool® and Permanet®. This was anticipated as nets with longer usage are frequently exposed to increased wear and tear through washing, scrubbing on hard surfaces, outdoor drying, and exposure to indoor smoke and heat. The findings are consistent with studies from Uganda [17], Liberia [25], India [26], Ethiopia [27], Cambodia [28], Zambia [29], Madagascar [30], and Tanzania [21, 23].

LLINs might be compromised before the insecticidal activity falls below established thresholds indicating the need for replacement [31, 32], and multiple reports have documented physical damage to nets under conditions of routine use. Various field trials have shown that Interceptor® nets are effective against malaria vectors in different countries and settings. Interceptor® LLIN was awarded in 2012 as a recommended LLIN based on phase I laboratory testing and phase II experimental hut studies [21]. In India, Interceptor® was reported to contain an average of 43.5 mg/m^2^ of residual insecticide after 3 years of field use [26], which is just above the level normally associated with effective vector control when using alpha-cypermethrin (40 mg/m^2^) in conventional treatment [33]. A study in Madagascar reported that 83.5%, 74%, and 68.5% of Netprotect®, Royal Sentry®, and Yorkool® nets, respectively, were physically damaged after 12 months of distribution [30].

During two long-term assessments of polyester-based LLINs, more than 70% of nets had holes after a year and more than 85% after 2 years in Uganda [17]. The mean insecticide levels declined by 25.9% from baseline of 66.2 + 14.6 mg/m^2^ at three to six months to 44.1 + 21.2 mg/m^2^ at 14–20 months and by 30.8% to 41.1 + 18.9 mg/m^2^ at 26–32 months in Ethiopia [27]. The WHOPES guidelines on monitoring the durability of LLINs outline methods are to estimate the hole sizes on the net fabric [15]. As the guidelines did not specify criteria for replacing expired nets, the WHO 80% cutoff of sampled LLINs that retain bio-efficacy [15] suggest that the median lifespan of Interceptor is probably between two to three years. Many studies in Africa have shown the wide variation in lifespan between individual nets and settings and assessed net failure based on a combination of variables reflecting attrition, physical integrity, and insecticidal effectiveness of nets. Using current but limited evidence, WHO has suggested calculating functional survival for both physical integrity and attrition of nets [34]. Also, estimates of insecticidal effectiveness for net durability in this study design were not available. The minimal effective concentration of insecticide in a net and its interpretation for bioassay results is unknown. In addition, current methods require removal and destruction of a small sub-sample of nets that adds complexity to evaluation. Prospective studies of insecticide effectiveness in a larger sample size of nets are required to draw strong conclusions.

### Malaria vectors in Nepal and the effectiveness of LLINs

A total of 44 species of *Anopheles* mosquitoes have been identified in Nepal based on the morphology; however, only seven species have been reported as malaria vectors which include *Anopheles minimus*, *Anopheles fluviatilis*, *Anopheles annularis*, *Anopheles maculatus*, *Anopheles dravidicus*, *Anopheles pseudowillmori*, *and Anopheles willmori* [35]*.* Over the last decades, deforestation and effective malaria control program using DDT eliminated *An. minimus* during 1960s [36]. *An. fluviatilis* is considered as the main malaria vector in Nepal followed by *An. annularis* and *An. maculatus* complex. Although LLINs are the essential tools for vector control and thus for malaria elimination, their effectiveness in South (East) Asia has been particularly compromised because of the early evening feeding time and preferentially outdoor biting of the *Anopheles* species [37, 38]. Nevertheless, globally, LLINs are recommended as an adjunctive tool among other approaches for malaria control and elimination [37, 38]. Modeling studies have suggested that an estimated 30% reduction in transmission of malaria can be achieved with the wide coverage of LLINs [39]. Thus, the distribution of LLINs with high coverage can become a critical adjunctive tool for the malaria elimination in Nepal.

### Implications for malaria control and elimination

National malaria control program in Nepal has a target to achieve the goal of eliminating malaria by 2025. To achieve the goal of malaria elimination, Nepal can utilize three-pillar strategies outlined by global technical strategy of World Health Organization that includes ensuring universal access to malaria prevention, diagnosis, and treatment; accelerating efforts towards elimination and attaining malaria-free status; and transforming malaria surveillance and response as core interventions [40]. The currently used tools for malaria elimination in Nepal can be informed by the effectiveness and durability of LLINs as evidenced from this study. A high coverage of effective LLIN may become an important adjunctive tool for malaria elimination in Nepal.

## Strengths and limitation

Since direct inter-LLIN comparison is invalid using a retrospective methodology, the performance of each LLIN brand is discussed separately. As Interceptor® has a relatively long history of distribution compared to the other two LLIN brands, it was possible to sample more than 50 nets after 3 years of its use in the community based on the WHO guidelines. During the first and second years post distribution, the sample size was less than 30 and zero, respectively, which limited the analysis of LLIN effectiveness; this was also seen in Yorkool® (in the second and third year) and PermaNet® 2.0 (first and third year). Our observations found that Interceptor® nets did not meet the WHO bioassay criteria as they have very low chemical retention (5.82%) and high physical damage, suggesting that its serviceable life was probably less than 3 years.

## Conclusion

Distribution of LLINs is effective for control of malaria but serviceable life of LLIN should be considered in terms of waning residual bio-efficacy that requires replacement. Monitoring of net use, fabric integrity, and loss of insecticidal capacity of various LLIN brands in routine intervals should be considered for effectiveness of LLINs for malaria control.

## Data Availability

The datasets used and/or analyzed during the current study are available from the corresponding author on reasonable request.

## Abbreviations

ANC: Antenatal care
CIPAC: Collaborative International Pesticide Analytical Council
CQ: Chloroquine
EDCD: Epidemiology and Diseases Control Division
GC-FID: Gas chromatography with flame ionization detection
HPLC-DAD: High performance liquid chromatography with UV diode array detection
IRS: Insecticidal residual spraying
KDR: Knock down rates
LLINs: Long-lasting insecticidal nets
MOHP: Ministry of Health and Population
NMCP: National Malaria Control Program
pHI: Proportionate hole index
PQ: Primaquine
RSD: Relative standard deviation
VDCs: Village Development Committees
WHO: World Health Organization
WHOPES: World Health Organization Pesticide Evaluation Schemes
